# Breeding Potential of Introgression Lines Developed from Interspecific Crossing between Upland Cotton (*Gossypium hirsutum*) and *Gossypium barbadense*: Heterosis, Combining Ability and Genetic Effects

**DOI:** 10.1371/journal.pone.0143646

**Published:** 2016-01-05

**Authors:** Jinfa Zhang, Man Wu, Jiwen Yu, Xingli Li, Wenfeng Pei

**Affiliations:** 1 Department of Plant and Environmental Sciences, New Mexico State University, Las Cruces, NM, United States of America; 2 State Key Laboratory of Cotton Biology, Institute of Cotton Research, Chinese Academy of Agricultural Sciences, Anyang, 455000, China; National Key Laboratory of Crop Genetic Improvement, CHINA

## Abstract

Upland cotton (*Gossypium hirstum* L.), which produces more than 95% of the world natural cotton fibers, has a narrow genetic base which hinders progress in cotton breeding. Introducing germplasm from exotic sources especially from another cultivated tetraploid *G*. *barbadense* L. can broaden the genetic base of Upland cotton. However, the breeding potential of introgression lines (ILs) in Upland cotton with *G*. *barbadense* germplasm integration has not been well addressed. This study involved six ILs developed from an interspecific crossing and backcrossing between Upland cotton and *G*. *barbadense* and represented one of the first studies to investigate breeding potentials of a set of ILs using a full diallel analysis. High mid-parent heterosis was detected in several hybrids between ILs and a commercial cultivar, which also out-yielded the high-yielding cultivar parent in F_1_, F_2_ and F_3_ generations. A further analysis indicated that general ability (GCA) variance was predominant for all the traits, while specific combining ability (SCA) variance was either non-existent or much lower than GCA. The estimated GCA effects and predicted additive effects for parents in each trait were positively correlated (at *P*<0.01). Furthermore, GCA and additive effects for each trait were also positively correlated among generations (at *P*<0.05), suggesting that F_2_ and F_3_ generations can be used as a proxy to F_1_ in analyzing combining abilities and estimating genetic parameters. In addition, differences between reciprocal crosses in F_1_ and F_2_ were not significant for yield, yield components and fiber quality traits. But maternal effects appeared to be present for seed oil and protein contents in F_3_. This study identified introgression lines as good general combiners for yield and fiber quality improvement and hybrids with high heterotic vigor in yield, and therefore provided useful information for further utilization of introgression lines in cotton breeding.

## Introduction

Heterosis, i.e., hybrid vigor, describes performance superiority of a hybrid over the average of its genetically distinct parents (i.e., mid-parent value) in morphological, physiological and biochemical characteristics such as body size, growth rate, fertility, and productivity. Heterosis is one of the most widely utilized biological phenomena in crop production and animal husbandry.

Upland cotton (*Gossypium hirsutum* L.) is the most important natural fiber crop in the world, and China is the largest cotton producer among more than 80 cotton-producing countries. Heterosis exists in cotton [1], and it has been extensively studied in China since the 1950s [2]. However, the commercialization of hybrid cotton did not start until the 1980s, and its production reached to more than 10% of the cotton acreage in China in the late 1990s [3, 4], which was doubled in the mid-2000s [5, 6]. Since then, most of the cotton hybrids carry a lepidoptera resistant Bt gene. Hybrid cotton seeds in China are predominantly produced through a hand emasculation and pollination process, while genetic and cytoplasmic male sterility systems [7, 8, 9, 10] have not been widely utilized in China. However, there are several issues in hybrid cotton research and production in China [6]. First, due to the fact that 90% of the hybrid cotton seeds were produced through hand emasculation, it is labor-intensive and time consuming, and therefore expensive. Second, the purity of hybrid seeds is questionable due to the lack of quality control measurements in hand emasculation and pollination. Third, most of the hybrids are only locally adapted and cannot be grown in different production regions and their yield stability varied among years and locations. Fourth, most of the hybrids do not produce superior fiber quality. Fifth, identifying high heterotic hybrids is difficult due to a lack of genetic diversity of parental lines.

Therefore, increasing genetic diversity in parental lines is vital to develop hybrid cottons with a high heterotic vigor in yield and good fiber quality. Several approaches can be taken for increasing genetic diversity in parents, such as (1) using Acala cotton which has the best fiber quality in Upland cotton and is known to have *G*. *barbadense* (known as Egyptian, Pima cotton, or Sea-Island cotton) germplasm introgression [11, 12], and/or (2) developing and using introgression lines through interspecific crossing and backcrossing between Upland and Pima cotton. Yu et al. [13] reported the development of a backcross inbred line (BIL) population and its use in quantitative trait locus (QTL) mapping. The BILs were developed through two generations of backcrossing followed by several generations of self- pollination. Only limited chromosomal regions from the *G*. *barbadense* donor parent were transferred to the recurrent parent through backcrossing, and repeated self-pollination also minimized hybrid breakdown and stabilized the chromosome segments transferred from *G*. *barbadense* to Upland cotton. Many BILs were found to have improved fiber quality in length, strength and micronaire than Upland cotton, some of which had similar lint yield to their recurrent Upland parent, indicating introgression of desirable fiber quality genes from Pima to Upland cotton. Therefore, these BILs may serve as a good source of parental lines in producing high heterotic hybrids with high yield potentials and good fiber quality.

To study the utility of germplasm in breeding, parental lines are crossed either in dialell design or factorial design (i.e., North Carolina Design II) to produce F_1_ hybrids for replicated evaluation in crops such as maize, rice and canola. In cotton, F_2_ progeny is often used for replicated evaluation due to the inefficiency of producing large quantity of F_1_ hybrid seeds through hand emasculation and pollination for testing in multiple environments [14, 15, 16, 17, 18, 19, 20, 21, 22]. It is unclear how efficient the F_2_ seeds are as the proxy for F_1_ although they have been used in these evaluations. The correlation between F_1_ and F_2_ in performance has not been well studied to establish a consistent relation between the two generations. Furthermore, breeding values of parental lines may need to be further evaluated in advanced generations such as F_3_, because the dominance effects and their interactions could confound the estimation of genetic parameters. The objectives of this study were to, (1) evaluate mid-parent heterosis and useful heterosis in F_1_, F_2_ and F_3_ generations from a full diallel cross of six selected introgression lines (ILs) and two commercial cultivars; (2) estimate general combining ability (GCA) and specific combining ability (SCA) variances and effects in the three generations; and (3) predict genetic effects in the parental lines and hybrids.

## Materials and Methods

In the current study, six introgression lines (NMGA-017, NMGA-085, NMGA-096, NMGA-098, NMGA-100, and NMGA-145), developed in New Mexico State University (Las Cruces, New Mexico, US), were selected from a total of 146 BILs [13] based on yield and fiber quality traits, together with two local Chinese commercial cultivars, CRI 44 and CRI 45, as parents. Fifty-six crosses were made among the eight parents in a full diallel design including reciprocals in 2006. The resulting F_1_ seeds were grown in the Experimental Farm, Institute of Cotton Research, Chinese Academy of Agricultural Sciences, Anyang, Henan province (hereafter AY), in 2007. A total of 64 entries including the 56 F_1_ hybrids and their 8 parents were arranged in a randomized complete block design with two replications. Seeds were sown directly to the field under plastic mulch in mid-April, and crop management followed the local recommendations. The plot length was 1-row × 8 m in length with a row spacing of 0.75 m and plant spacing of 0.23 m, and seedlings were thinned to 32 plants plot^-1^. In 2008, the same test using the same experimental design was grown in the same location for the 56 F_2_ progeny together with the 8 parents. In 2009, the 56 F_3_ progeny together with the 8 parents were sown in the same location and two other locations (Huiming, Shandong province, hereafter SD; and Shangqiu, Henan province, hereafter SQ). The experimental design was the same as above with also two replications in each location. The tests were managed as per local recommendations for each location.

At crop maturity, 25 open boll samples per plot (1 boll from the middle of the plants per plant) were hand harvested and ginned in a 10 saw laboratory gin, and seedcotton and lint were weighed to estimate boll weight (BW, g of seedcotton boll^-1^) and lint percent (LP, % of seedcotton weight accounted by lint weight). A subsample of the lint in each plot was evaluated for fiber quality traits using the High Volume Instrument (HVI) 900 (Test Center of Cotton Fiber Quality affiliated with the Agriculture Ministry of China, Institute of Cotton Research, Chinese Academy of Agricultural Science, Anyang, Henan province, China). The fiber quality traits measured were fiber length (FL), fiber strength (FS), and micronaire (MIC). Individual plots were then hand harvested for determination of the seedcotton weight which was converted to seedcotton yield (SCY, kg ha^-1^). Lint yield (LY, kg ha^-1^) was then estimated by multiplying SCY with the lint percent estimated from the 25 boll sample ginned. A subsample of seed from each ginned boll sample was determined for gossypol, oil and protein contents using the methods as described by Yu et al. [23].

The results from above field tests were first subjected to a combined analysis of variance using SAS (SAS Institute 2000). Only the traits with significant genotypic variances were further analyzed for general combining ability (GCA) of parents and specific combining ability (SCA) for the hybrids based on Griffing’s Method 1 Model 1 [24] using DIALLEL-SAS05 developed by Zhang et al. [25]. Mid-parent heterosis (MPH) was estimated as a percentage of the difference between the mean of F_1_, F_2_ or F_3_ and mid-parent mean divided by the mid-parent mean. The mid-parent mean was calculated as the average of both parents with respect to a trait of interest. Useful heterosis in LY was also estimated as a percentage of the difference between the mean of F_1_, F_2_ or F_3_ and the mean of the highest-yielding parent (i.e., CRI 44) in each test.

To estimate all variance components, the results from the three hybrid generations were further analyzed using a linear mixed model approach, i.e., minimum norm quadratic unbiased estimation (MIQNUE) [26]. Additive and dominance effects were predicted by the adjusted unbiased prediction (AUP) method based on the additive-dominance-maternal (ADM) genetic model [27]. The data were analyzed using the QGA software (http://ibi.zju.edu.cn/software/qga/index.htm).

## Results

### Analysis of variance

In this study, 6 introgression lines (ILs) and 2 commercial cultivars were crossed to generate 56 F_1_ hybrids in a full diallel. The F_1_ and F_2_ hybrids were tested with their parents at one location in 2007 and 2008, respectively, and the 56 F_3_ and the 8 parents were also tested at three locations in 2009. Therefore, analyses of variance were performed for the three hybrid generations separately, and the results are shown in Table 1 for seedcotton yield, lint yield, yield components, fiber quality, and seed quality traits.

**Table 1:** Analyses of variance on combining ability of yield, yield components, and fiber and seed quality traits based on a full diallel of 8 parents (6 introgression lines and 2 commercial cultivars) in their 56 F_1_, 56 F_2_, and 56 F_3_ hybrids of Upland cotton.

S.O.V	DF	SCY	LY	LP	BW	FL	FS	MIC	GOS	OIL	PRO
**F**_**1**_											
Replicationl	1	16.8	21.5	3.35	0.13	0.68	4.31*	0.14			
Genotype	63	2183.6*	478.1**	7.97**	0.25*	1.22**	1.12	0.09*			
GCA	7	6613.6**	1932.8**	52.76**	0.96**	5.36**	2.39*	0.48**			
SCA	28	2269.8	425.6*	3.13	0.22	0.71*	0.9	0.04			
Reciprocal	28	989.9	166.8	1.6	0.08	0.69*	1.04	0.04			
Error	63	1095.1	173.8	2.2	0.13	0.37	0.8	0.04			
**F**_**2**_											
Replication	1	10633.0**	1837.9**	2.16	0.03	0.68	0.52	0.08			
Genotype	63	2233.0**	446.8**	7.57**	0.16**	0.85*	1.75**	0.06*			
GCA	7	13632.2**	2829.8**	47.01**	0.84**	3.60**	8.07**	0.29**			
SCA	28	859.8	158.0*	3.04*	0.08	0.5	1.169*	0.04			
Reciprocal	28	756.3	139.7	2.25	0.06	0.52	0.75	0.03			
Error	63	535.9	87.7	1.312	0.05	0.38	0.63	0.03			
**F**_**3**_											
Environment (E)	2	7862908**	1385295**	185.49**	83.03**	395.10**	476.66**	4.14**	0.11**	42.69**	128.77**
Replication	3	24531.6**	8562.1**	22.65**	0.08	5.77**	1.31	0.18**	0.13**	9.64**	4.06**
Genotype (G)	63	5304.1**	1489.5**	10.20**	1.02**	1.64**	2.69*	0.18**	0.02**	38.41**	32.94*
G × E	126	1547.9*	434.8**	2.36*	0.27**	0.44	1.33**	0.06**	0.002	2.04**	1.21**
GCA	7	23025.2**	7402.7**	64.99**	5.60*	8.83**	14.65**	1.05**	0.10**	278.51**	243.99**
SCA	28	4518.5**	1117.4**	4.82**	0.42*	0.80*	1.30*	0.07*	0.008**	13.67**	10.76**
GCA × E	7	4782.7**	1860.1*	5.82*	0.69	0.59	4.03**	0.34*	0.007*	5.33*	1.146**
SCA × E	28	1498.9	448.2	1.83	0.29	0.31	0.87	0.06	0.002	1.62	1.13
Reciprocal (Rec)	28	1659.4*	383.3*	1.88	0.47	0.69	1.04	0.07*	0.002	3.12*	2.370**
Rec × E	28	1866.4	407.9	1.37	0.31	0.7	1.45	0.04	0.001	1.239	0.58
Error	189	929.9	211	1.67	0.25	0.41	0.8	0.05	0.002	0.83	0.67

*SCY* seedcotton yield, *LY* lint yield, *LP* lint percent, *BW* boll weight, *FL* fiber length, *FS* fiber strength, *MIC* micronaire, *GOS* seed gossypol content, *OIL* seed oil content, *PRO* seed protein content, *GCA*, general combining ability, *SCA*, specific combining ability

*P<0.*05*,

**P<0.*01*.

Significant variations due to genotype (G) and significant GCA effects were detected for all the traits evaluated in all the three hybrid generations. For SCA, significant variances were detected for lint yield and fiber length in F_1_, for lint yield, lint percent and fiber strength in F_2_, and for all the traits in F_3_ (Table 1). In F_3_, significant GCA × E (i.e., environment) were also detected for seedcotton and lint yields, fiber strength, micronaire, and seed gossypol, oil and protein contents; however, no SCA × E was detected. Except for micronaire in F_1_, no significant variances for reciprocal effects were detected in F_1_ and F_2_; however, they were detected for seedcotton yield, lint yield, micronaire, seed oil, and protein contents in F_3_. Because F_1_, F_2_, and F_3_ were tested in different tests or years, there were no interactive effects of combining ability with generation. Therefore, these effects were not analyzed.

### Parent performance

The results for parent performance tested in three years are shown in Table 2. The two commercial cultivars CRI 44 and CRI 45 had the highest and similar seedcotton yield (SCY) among the eight parents while the six introgression lines (ILs) produced 79–85% of SCY of CRI 44. CRI 44 had the highest LP (41.9%), followed by CRI 45 and NMGA-096 (40.1%), while other five ILs had LP ranging from 36.9 to 39.3%. Due to lower lint percent (LP), the six ILs produced 69–76% of lint yield (LY) of CRI 44, and CRI 45 yielded 94% of CRI 44.

**Table 2:** Parent performance in yield, yield components, fiber quality, and seed quality traits across five field tests (Anyang, 2007, 2008, and 2009; Shangdong, 2009; and Sangqiu, 2009).

Parent	SCY	LY	LP	BW	FL	FS	MIC	GOS	OIL	PRO
	kg ha^-1^	kg ha^-1^	%	g boll^-1^	mm	cN tex^-1^	unit	%	%	%
NMGA-017	3518	1357	37.73	6.07	30.34	30.05	4.51	0.89	27.69	36.52
NMGA-085	3411	1319	37.97	5.26	29.08	28.47	4.74	1.02	30.25	33.63
NMGA-096	3307	1341	40.19	5.92	30.10	29.38	4.36	0.85	27.73	36.37
NMGA-098	3272	1281	38.80	5.15	28.52	28.11	4.89	0.92	30.31	33.80
NMGA-100	3274	1212	36.89	5.31	29.26	28.55	4.75	0.93	30.50	33.56
NMGA-145	3353	1335	39.33	5.18	29.40	29.25	4.38	0.88	28.19	35.86
CRI 44	4163	1756	41.92	5.66	29.25	28.04	4.49	0.82	23.64	39.45
CRI 45	4041	1647	40.54	5.62	29.22	28.90	4.89	0.87	24.65	39.06

*SCY* seedcotton yield, *LY* lint yield, *LP* lint percent, *BW* boll weight, *FL* fiber length, *FS* fiber strength, *MIC* micronaire, *GOS* seed gossypol content, *OIL* seed oil content, *PRO* seed protein content

For fiber length, both commercial cultivars were similar, 29.2 and 29.3 mm, respectively, which were also similar to that of NMGA-085, NMGA-100, and NMGA-145 (29.1–29.4 mm). Two ILs, i.e., NMGA-17 and NMGA-096, had significantly longer fiber length, 30.1 and 30.3 mm, respectively. NMGA-017 also had the strongest fiber strength (30.1 cN tex^-1^), while other lines had strength ranging from 28 to 29.4 cN tex^-1^. The six ILs had similar micronaire to the two commercial cultivars (4.4–4.9).

For seed quality traits, NMGA-085 had the highest gossypol content (1.02%), while other seven genotypes were similar (0.82–0.93%). Interestingly, the two commercial cultivars had the lowest seed oil content (23.6–24.7%), while all of the six ILs had higher oil contents including three lines at 27.7–28.2% and the other three ILs at 30.2–30.5%. However, the two commercial lines had the highest protein contents (39.1–39.5%), significantly higher than the six ILs. Among the six ILs, three lines had the lowest protein contents (33.6–33.8%), and the other three were intermediate (35.9–36.4%).

### General and specific combining ability

GCA effects were estimated for F_1_, F_2_, and F_3_ generations separately (Table 3). Not surprisingly, the two commercial cultivars had the highest and positive GCA effects for SCY, LY, and LP in the three generations, while three ILs (NMGA-017, NMGA-085, and NMGA-098) had the lowest and negative GCA effects for SCY and LY. NMGA-017 and NMGA-100 also had lowest GCA effect for LP in F_1_ and F_2_. NMGA-017, NMGA-096, and CRI 44 had higher GCA effects for boll weight, while NMGA-098 and NMGA-145 had lowest and negative effects for the same trait.

**Table 3:** General combining ability (GCA) for yield, yield component traits and fiber quality traits in 6 introgression lines and 2 commercial Upland cultivars based on a full diallel of 56 F_1_, F_2_ and F_3_ hybrids of Upland cotton (*Gossypium hirsutum*).

Parents (ID)	SCY	LY	LP	BW	UHM	STR	MIC	GOY	OIL	PRO
**F1**										
NMGA-017 (P1)	-13.07*	-8.281*	-1.69**	0.25**	0.83**	0.37*	-0.03			
NMGA-085 (P2)	-13.66*	-6.50*	-0.54*	-0.156*	-0.148	0.08	-0.001			
NMGA-096 (P3)	-4.75	-0.139	0.96*	0.13*	0.162	-0.07	-0.12*			
NMGA-098 (P4)	-11.63*	-5.63*	-0.49*	-0.15*	-0.61**	-0.2	0.11*			
NMGA-100 (P5)	6.57	0.27	-1.12**	0.02	-0.09	-0.13	0.07*			
NMGA-145 (P6)	-4.74	-2.77	-0.43	-0.22*	-0.21*	0.31*	-0.18**			
CRI 44 (P7)	23.14**	13.29**	2.02**	0.156*	0.02	-0.45*	-0.02			
CRI 45 (P8)	18.14**	9.77**	1.31**	-0.04	0.05	0.1	0.18**			
**F2**										
NMGA-017 (P1)	-11.77*	-5.58*	-0.61*	0.19**	0.61**	0.90**	-0.05			
NMGA-085 (P2)	-1.65	-1.94	-0.88**	-0.07	-0.14*	-0.09	0.05			
NMGA-096 (P3)	-26.31**	-9.96**	0.59*	0.19**	0.32	0.29*	-0.16**			
NMGA-098 (P4)	-14.86*	-6.65**	-0.64*	-0.16**	-0.39*	-0.61**	0.14**			
NMGA-100 (P5)	-2.48	-2.63	-1.21**	-0.09	-0.01	-0.31*	0.07*			
NMGA-145 (P6)	-4.45	-2.43	-0.55*	-0.15*	-0.35	-0.14	-0.05*			
CRI 44 (P7)	28.92**	15.03**	2.40**	0.18**	0.001	-0.42*	-0.02			
CRI 45 (P8)	32.62**	14.18**	0.90**	-0.08*	-0.04	0.40*	0.04			
**F3**										
NMGA-017 (P1)	1.74	-1.28	-0.44*	0.42**	0.51**	0.48**	-0.02	-0.02**	-0.03	0.45**
NMGA-085 (P2)	-13.49**	-7.86**	-0.41*	-0.02	-0.19*	-0.25*	0.07*	0.06**	1.359**	-1.32**
NMGA-096 (P3)	-7.07*	-1.66	0.3	0.24**	0.41**	0.46**	-0.16**	0.001	0.92**	-0.74**
NMGA-098 (P4)	-4.301	-3.53*	-0.54**	-0.27**	-0.31**	0.04	0.08**	0.02**	1.157**	-1.12**
NMGA-100 (P5)	-5.164	-5.15*	-1.05**	-0.06	-0.22*	-0.13	0.03	0.21**	1.18**	-1.35**
NMGA-145 (P6)	-17.84**	-7.63**	-0.1	-0.30**	-0.19*	-0.07	-0.11**	0.002	0.730**	-0.72**
CRI 44 (P7)	25.88**	15.56**	1.53**	0.02	-0.002*	-0.71**	-0.04	-0.04**	-2.96**	2.58**
CRI 45 (P8)	20.25**	11.57**	0.72**	-0.03	0.004	0.19	0.13**	-0.03**	-2.35**	2.22**
GCA (F1/F2)	0.838**	0.865**	0.920**	0.912**	0.939**	0.755*	0.694			
GCA (F1/F3)	0.810*	0.883**	0.918**	0.876**	0.885**	0.912**	0.609			
GCA (F2/F3)	0.784*	0.853**	0.978**	0.861**	0.934**	0.859**	0.727*			
GCA-P: F1	0.807*	0.854**	0.942**	0.902**	0.899**	0.809*	0.933**			
GCA-P: F2	0.903**	0.902**	0.943**	0.899**	0.892**	0.884**	0.892**			
GCA-P: F3	0.919**	0.948**	0.963**	0.931**	0.923**	0.786*	0.958**			

*SCY* seedcotton yield, *LY* lint yield, *LP* lint percent, *BW* boll weight, *FL* fiber length, *FS* fiber strength, *MIC* micronaire, *GOS* seed gossypol content, *OIL* seed oil content, *PRO* seed protein content

*P<0.*05*,

**P<0.*01*.

For fiber quality traits (Table 3), NMGA-017 had the highest GCA effects for fiber length and strength in the three generations, while NMGA-098 and NMGA-145 had the lowest GCA for fiber length and micronaire. CRI 44 had consistent and lower GCA for strength, while CRI 45 had high GCA for micronaire.

Interestingly, except for GCA in F_1_ and F_2_ for micronare, GCA of parents was significantly or highly significantly and positively correlated with their performance for all the traits (Table 3). The results indicate that GCA is largely determined by the performance of parental lines. Furthermore, GCA among the three generations was significantly or highly significantly and positively correlated for each trait, indicating a high congruence in GCA estimates using different hybrid populations.

For all the traits (except for fiber length and seed protein content) that had significant GCA × E interactions in F_3_, GCA effects were estimated for each testing location (Table 4). The results were highly similar to the overall GCA effects as estimated across locations (Table 3). Except for boll weight, fiber strength, and micronaire in correlations between locations AY and SD, and between locations SD and SQ, GCA effects for each trait were significantly or highly significantly correlated between locations, indicating an overall high congruence between locations. For example, for lint yield with GCA × E, the two commercial cultivars had significant and positive GCA, while NMGA-085 had significant and negative GCA in all three locations. The trends were also similar for other parental lines, even though the magnitudes differed among locations. The same is true for other traits (Table 4). Because of the consistency of the results across locations, no further analysis was performed.

**Table 4:** General combining ability (GCA) for yield, yield component traits, and fiber quality traits in 6 introgression lines and 2 commercial Upland cultivars based on a full diallel of 56 F_3_ hybrids (*Gossypium hirsutum*) for 3 locations.

Trait	Loc	NMGA-017 (P1)	NMGA-085 (P2)	NMGA-096 (P3)	NMGA-098 (P4)	NMGA-100 (P5)	NMGA-145 (P6)	CRI 44 (P7)	CRI 45 (P8)	Loc	Correlation
SCY	AY 09	-2.770	-9.48*	-3.12	-8.2	-4.52	-5.35	17.47**	15.99**	AY09-SD09	0.863
	SD09	9.730	-18.25*	-12.97	-5.07	-5.69	-38.06**	36.74**	33.59**	AY09-SQ09	0.910
	SQ09	-1.730	-12.73*	-5.13	0.37	-5.27	-10.10*	23.43**	11.18*	SD09-SQ09	0.904
LY	AY 09	-1.930	-4.13*	-0.65	-4.28*	-3.26*	-2.32*	10.18**	6.41**	AY09-SD09	0.939
	SD09	-0.930	-14.90**	-2.38	-5.67	-7.53*	-16.16**	24.79**	22.80**	AY09-SQ09	0.944
	SQ09	-0.970	-4.54*	-1.96	-0.64	-4.66*	-4.41*	11.70**	5.51*	SD09-SQ09	0.945
LP	AY 09	-0.49*	-0.31	0.35*	-0.66*	-0.96**	-0.14	1.99*	0.22	AY09-SD09	0.955
	SD09	-0.260	0.19	0.11	-0.09	-0.86*	-0.08	1.74**	0.33	AY09-SQ09	0.837
	SQ09	-0.090	0.26	0.05	-0.34	-1.26**	-0.15	1.01**	0.52	SD09-SQ09	0.907
BW	AY 09	0.38**	-0.20**	0.19*	-0.19**	-0.04	-0.20**	0.07	-0.008	AY09-SD09	0.689
	SD09	0.43*	0.23	0.11	-0.42*	0.09	-0.35*	0.007	-0.1	AY09-SQ09	0.898
	SQ09	0.44**	-0.10*	0.43**	-0.19*	-0.24**	-0.36**	0.002	0.01	SD09-SQ09	0.673
STR	AY 09	0.008	-0.05	-0.21**	0.07	0.008	-0.12*	0.041	0.2**	AY09-SD09	0.520
	SD09	-0.070	0.13**	-0.11*	0.15**	0.08*	-0.095*	-0.10*	0.003	AY09-SQ09	0.806
	SQ09	0.010	0.13**	-0.186**	0.02	-0.006	-0.11*	-0.06*	0.20**	SD09-SQ09	0.546
MIC	AY 09	0.39*	-0.14	0.76**	0.14	-0.21	0.25	-1.25**	0.07	AY09-SD09	0.373
	SD09	0.72**	-0.18	0.042	-0.15	-0.01	-0.35*	-0.47*	0.41	AY09-SQ09	0.788
	SQ09	0.230	-0.43*	0.59**	0.147	-0.18	-0.09	-0.40*	0.12	SD09-SQ09	0.599
GOS	AY 09	-0.02**	0.04**	-0.004	0.015*	0.02*	0.002	-0.02**	-0.02*	AY09-SD09	0.954
	SD09	-0.02*	0.08**	-0.003	0.02*	0.02*	0.002	-0.06**	-0.042**	AY09-SQ09	0.909
	SQ09	-0.03*	0.04**	0.008	0.019*	0.01	0.003	-0.04**	-0.01	SD09-SQ09	0.916
OIL	AY 09	0.170	1.03**	0.79**	1.06**	1.28**	0.86**	-3.00**	-2.22**	AY09-SD09	0.986
	SD09	-0.210	1.95**	0.90**	1.61**	1.34**	1.01**	-3.55**	-3.05**	AY09-SQ09	0.983
	SQ09	-0.050	1.07**	1.05**	0.78**	0.93**	0.32*	-2.34**	-1.77**	SD09-SQ09	0.982

*SCY* seedcotton yield, *LY* lint yield, *LP* lint percent, *BW* boll weight, *FL* fiber length, *FS* fiber strength, *MIC* micronaire, *GOS* seed gossypol content, *OIL* seed oil content, *PRO* seed protein content

*P<0.*05*,

**P<0.*01*.

For SCA, only lint yield was found to be significant in three generations, and lint percent, fiber length and strength were significant in two generations. Other traits had significant SCA only in F_3_ (S1 Table). However, there was no correlation in SCA for the same trait between generations. SCA in lint yield was highly significantly and positively correlated with SCA in lint percent in F_2_ and F_3_, and with seed oil content in F_3_. But its correlation was highly significant and negative with SCA in seed protein content in F_3_. Furthermore, in F_3_ tested in three locations, no SCA × E was detected.

### Mid-parent heterosis in yield, yield components, and fiber quality and seed quality traits

The results are shown in Table 5. In F_1_, no negative mid-parent heterosis (MPH) for SCY and LY was detected, while the highest MPH reached 62.96–65.68%, with an average MPH of 31.36–32.00% across the 56 hybrids. In F_2_, inbreeding depression was noted in that some hybrids displayed the highest negative MPH at -27.25 to -28.70%, and the highest positive MPH was 70.77–81.24% with an average of 20.94–25.17%.

**Table 5:** Mid-parent heterosis of yield, yield components, fiber quality, and seed quality traits in F_1_ (2007), F_2_ (2008), and F_3_ (2009) from a full diallel cross of hybrids made from six introgression lines and two commercial cultivars.

Trait		F_1_	F_2_		F_3_	
		AY 07	AY 08	AY 09	SD 09	SQ 09
Seedcotton yield	Min	-1.06	-28.7	-10.93	-14.57	-12.12
	Max	61.96	70.76	50.51	32.02	32.33
	Mean	31.36	20.94	21.14	9.54	10.09
Lint yield	Min	-1.93	-27.25	-17.19	-17.15	-10.87
	Max	65.68	81.24	50.06	45.06	35.56
	Mean	32.00	25.17	22.72	12.70	10.08
Lint percent	Min	-8.11	-2.14	-4.80	-6.50	-7.21
	Max	6.79	15.93	6.27	10.83	10.19
	Mean	0.59	3.80	1.59	2.80	0.64
Boll weight	Min	-2.75	-12.18	-10.93	-14.48	-9.29
	Max	22.88	15.93	21.99	33.15	13.23
	Mean	8.95	-1.30	7.35	6.32	0.99
Fiber length	Min	-2.84	-5.68	-3.32	-3.16	-3.16
	Max	7.13	4.63	4.97	11.35	4.76
	Mean	2.46	0.02	1.67	3.08	0.86
Fiber strength	Min	-4.46	-6.00	-7.69	-6.66	-4.53
	Max	7.34	5.63	5.55	6.66	7.91
	Mean	0.93	-1.98	1.78	0.59	0.72
Micronaire	Min	-6.52	-6.13	-14.40	-12.25	-9.53
	Max	12.59	10.64	7.06	11.65	6.28
	Mean	1.45	0.64	-0.57	0.21	-0.55
Gossypol content	Min			-13.66	-12.18	-9.30
	Max			11.88	11.13	12.39
	Mean			0.21	-1.04	-0.06
Seed oil content	Min			-10.26	-13.44	-9.25
	Max			16.18	16.68	6.83
	Mean			-1.25	-1.41	-1.09
Seed protein content	Min			-9.38	-8.78	-5.66
	Max			7.25	8.40	7.54
	Mean			0.74	0.95	0.93

The F_3_ progenies of the 56 hybrids were further tested in the same location as the F_1_ and F_2_, in addition to two other locations. Although the MPH ranges for SCY (-10.93 to 50.51%) and LY (-17.19 to 50.06%) in Anyang were narrower, the mean MPH (21.14–22.72%) was similar to that for F_2_ (Table 5). However, the mean MPHs from the other two locations were much lower (9.54–12.70% at SD and 10.08–10.09% in SQ). Therefore, further inbreeding depression in productivity was observed in F_3_.

The MPH for lint percent ranged from -8.11 to 6.79% in F_1_ and -2.14 to 15.93% in F_2_, in Anyang, and -4.80 to 10.83% in F_3_ in three locations (Table 5). On average, very little MPH (0.59 to 3.8%) and no apparent inbreeding depression were observed. However, for boll weight, it did not display any negative MPH while its positive MPH reached 22.88% with an average of 8.95% in F_1_ (Table 5). In F_2_, negative MPH was noted with an average MPH of -1.30%. Similar MPH ranges were observed in F_3_, with an overall positive MPH (6.32–7.35%) in two locations.

For fiber quality traits, the MPH for individual hybrids ranged from-5.68 to 11.35% for fiber length, -7.69 to 7.91% for strength, and -12.25 to 12.59% for micronaire in F_1_, F_2_ and F_3_. The overall MPH and inbreeding depression were low to negligible (Table 5). The hybrid across generations and tests with the best fiber quality was NMGA-96 × NMGA-17, which showed transgressive segregation in fiber length and strength, even though NMGA-17 had the longest and strongest fibers among the eight parents.

Seed quality was determined in the F_3_ progeny of the 56 hybrids and their eight parents in three locations (Table 5). No apparent overall MPH was detected for seed gossypol content, while the overall MPH for seed oil content was small but negative (-1.09 to -1.41%), and it was small but positive for seed protein content (0.74–0.95%).

### Best performing hybrids (useful heterosis) in lint yield

Since lint yield is the most important trait for evaluating the possible potential of a hybrid in cotton production, top yielders were further analyzed. Among F_1_ tested in 2007, 14 over-yielded CRI 44 in lint by 10.05–34.22%, including reciprocal hybrids of CRI 44 × NMGA-100, CRI 44 × NMGA-145, CRI 45 × NMGA-98, and CRI 45 × NMGA 145 (S2 Table). NMGA-145 appeared to be a good IL to be used in producing high heterotic hybrids. The results indicate that the best hybrids with high heterotic vigors had one commercial cultivar as the parent, and reciprocal hybrids performed similarly. On the other hand, all the low-yielding hybrids had ILs as both of their parents. The reciprocal hybrids of CRI 44 × CRI 45 only performed well in one test (i.e., AY, 2008).

Although there was an overall congruence between F_1_ and F_2_ performance in lint yield (r = 0.430, *P<0*.*01*, Table 5), only 3 of the 14 top yield performers in F_1_ were among the top six high yielders in F_2_ tested in the same location but different year. However, the results in F_3_ from three locations were more consistent with F_1_ in that, out of the top 14 high yielders in F_1_, 8–10 were also among the top yielders in F_3_ tested in all the three locations.

Across generations and locations, CRI 44 × NMGA-100 was the best hybrid cross combination, with useful heterosis over CRI 44 (one of the two commercial parents with higher yield) at 25.29–33.69% in F_1_ and -11.14 to 9.01% in F_2_. The useful heterosis was also detected in F_3_ with 6.93–11.63% in AY, 0.35–18.87% in SD, and 3.10–14.25% in SQ.

### Correlations between mid-parent heterosis for lint yield and other traits and correlations of MPH among F_1_, F_2_ and F_3_

The results are shown in Table 6. As lint yield is the product of SCY and lint percent, and SCY is the product of boll weight × number of harvested bolls, the most significant and consistent correlation was between lint yield and SCY, followed by the correlation between lint yield and lint percent in all the generations across all the tests. Lint yield was also significantly correlated with boll weight in two tests (F_1_ and F_3_ in Anyang 2007 and 2009). Unexpectedly, lint yield was correlated with fiber length, strength and micronaire in F_3_ in only one test (AY, 2009) and with fiber length in another (SD, 2009). However, it was consistently correlated with the three seed quality traits determined in F_3_ in all the three tests in that its correlations with seed gossypol and oil contents were negative, but positive with seed protein content (Table 6).

**Table 6:** Coefficients of correlation between mid-parent heterosis (MPH) for lint yield and MPH for other traits and between lint yield and other traits in F_1_ (2007), F_2_ (2008), and F_3_ (2009) from a full diallel cross of hybrids made from six introgression lines and two commercial cultivars.

Trait	F_1_	F_2_		F_3_	
	AY 07	AY 08	AY 09	SD 09	SQ 09
**Hybrid performance**					
Seedcotton yield	0.974**	0.989**	0.976**	0.953**	0.975**
Lint percent	0.663**	0.659**	0.705**	0.794**	0.582**
Boll weight	0.332*	0.081	0.394**	0.119	0.174
Fiber length	0.184	-0.005	0.285*	0.353**	0.106
Fiber strength	-0.154	-0.048	-0.280*	0.135	-0.075
Micronaire	0.194	0.114	0.268*	-0.075	0.079
Gossypol content			-0.455**	-0.599**	-0.545**
Seed oil content			-0.733**	-0.786**	-0.751**
Seed protein content			0.711**	0.766**	0.766**
**MPH**					
Seedcotton yield	0.970**	0.991**	0.972**	0.957**	0.959**
Lint percent	0.419**	0.262	0.523**	0.742**	0.468**
Boll weight	0.032	0.197	0.401**	0.050	0.135
Fiber length	0.209	0.172	0.330*	0.312*	-0.116
Fiber strength	-0.092	0.041	0.221	0.032	-0.192
Micronaire	-0.001	0.023	0.132	0.274*	0.166
Gossypol content			-0.242	-0.279*	-0.452**
Seed oil content			-0.354**	-0.629**	-0.537**
Seed protein content			0.362**	0.566**	0.594**

*P<0.*05*,

**P<0.*01*.

Similar results were obtained for correlations in MPH between lint yield and other traits (Table 6). MPH for SCY was highly correlated with MPH for lint yield in all tests, as expected, and the correlation between lint yield and lint percent was significant in all tests except for F_2_ in AY, 2008. The MPH for lint yield was also significantly correlated with fiber length and micronaire in 1–2 tests. The MPH for lint yield and MPH for the three seed quality traits had similar correlations as the hybrid performance per see in all the tests except for seed gossypol content in F_3_ tested in AY, 2009.

As shown in Table 7, hybrid performance in F_1_, F_2_ and F_3_ were all positively and significantly correlated except for fiber strength between F_1_ and F_2_ tested in AY, 2007, and in F_3_ between SD and SQ. The results indicate that hybrid performance were overall congruent in the three generations and across the three testing locations in F_3_. However, there was no correlation in MPH for all the traits among the three generations tested in AY, 2007–2009. Interestingly, MPH in F_3_ had better correlations among the three locations in lint yield and the three seed quality traits, and the correlations in MPH for other traits among locations were mostly consistent (Table 7).

**Table 7:** Correlation coefficients of traits for MPH and hybrid performance among F_1_, F_2_ and F_3_ tested in the Anyang location and among locations in F_3_.

Trait	F_1_/F_2_	F_1_/F_3_	F_2_/F_3_	AY/SD	AY/SQ	SD/SQ
**Hybrid performance**						
Seedcotton yield	0.430**	0.581**	0.410**	0.595**	0.528**	0.518**
Lint yield	0.536**	0.668**	0.500**	0.718**	0.619**	0.593**
Lint percent	0.794**	0.679**	0.622**	0.612**	0.470**	0.366**
Boll weight	0.536**	0.596**	0.526**	0.462**	0.766**	0.437**
Fiber length	0.503**	0.390**	0.475**	0.443**	0.491**	0.458**
Fiber strength	0.107	0.297*	0.352**	0.329*	0.351**	0.112
Micronaire	0.436**	0.536**	0.286**	0.334**	0.558**	0.320*
Gossypol content				0.676**	0.643**	0.690**
Seed oil content				0.929**	0.933**	0.890**
Seed protein content				0.943**	0.939**	0.908**
**MPH**						
Seedcotton yield	0.010	0.233	-0.239	0.316*	0.208	0.358**
Lint yield	-0.037	0.252	-0.237	0.365**	0.312*	0.367**
Lint percent	0.392*	0.177	0.062	0.210	0.253*	0.009
Boll weight	-0.142	0.082	-0.158	0.120	0.569**	0.202
Fiber length	0.241	0.022	0.263	0.192	0.194	-0.094
Fiber strength	0.220	-0.002	0.154	0.208	-0.067	-0.090
Micronaire	0.433**	0.120	-0.029	-0.055	0.324*	-0.117
Gossypol content				0.334*	0.467**	0.421**
Seed oil content				0.779**	0.710**	0.638**
Seed protein content				0.826**	0.696**	0.673**

*P<0.*05*,

**P<0.*01*.

### Partition of genetic variance, estimation of genetic effects and their correlation with combining ability

Genetic variance was further partitioned into individual variance components for each trait in each test using a minimum norm quadratic unbiased estimation (MIQNUE)-based ADM model, and the results are shown in Table 8. Additive variance (*V*_*A*_), dominance variance (*V*_*D*_), and maternal variance (*V*_*M*_) were detected for all the traits in F_1_, except for *V*_*M*_ in boll weight and micronaire. Similarly, *V*_*A*_ was detected for all the traits in F_2_. However, *V*_*D*_ was not significant for SCY, lint yield (LY), lint percent (LP), and fiber length, while *V*_*M*_ was detected only for these four traits. In F_1_, both *V*_*A*_ and *V*_*D*_ were equally important, while *V*_*A*_ was predominant for traits other than the above four traits. In F_2_, *V*_*A*_ was predominant for these four traits, while *V*_*D*_ was predominant for other three traits (i.e., BW, FS and MIC).

**Table 8:** Estimates of variance components based on a complete diallel set of 56 F_1_ and F_2_ hybrids together with their eight parents (six introgression lines and two commercial cultivars) of Upland cotton tested in Anyang, 2007 and 2008.

Parameter	SCY	LY	LP	BW	FL	FS	MIC
**F**_**1**_							
*V*_*A*_	261.8**	88.4**	2.279**	0.042**	0.423**	0.153**	0.036**
*V*_*D*_	439.4**	88.2**	0.345**	0.041**	0.083**	0.017**	0.000**
*V*_*M*_	129.6**	22.3**	0.043**	0.000	0.000**	0.124**	0.000
*V*_*E*_	979.7**	157.5+	2.027**	0.118**	0.470**	0.835**	0.042**
*V*_*P*_	1810.5**	356.4**	4.694**	0.201**	0.976**	1.130**	0.078**
*V*_*A*_*/V*_*p*_	0.145**	0.248**	0.485**	0.210**	0.433**	0.136**	0.457**
*V*_*D*_*/V*_*p*_	0.243**	0.247**	0.073**	0.203**	0.085**	0.015	0.002
*V*_*M*_*/V*_*p*_	0.072**	0.062**	0.009	0.000	0.000	0.110**	0.000
*V*_*e*_*/V*_*p*_	0.541**	0.442**	0.432**	0.587**	0.482**	0.739**	0.541**
*H*_*N*_^*2*^	0.216**	0.311**	0.495**	0.210**	0.433**	0.246**	0.457**
*H*_*B*_^*2*^	0.459**	0.558**	0.568**	0.413**	0.518**	0.261**	0.459**
**F**_**2**_							
*V*_*A*_	996.4**	212.3**	3.583**	0.020**	0.206**	0.175**	0.007**
*V*_*D*_	0.000	0.000	0.000	0.144**	0.000	1.378**	0.048**
*V*_*M*_	1305.3**	300.7**	6.370**	0.000	0.126**	0.000	0.000
*V*_*E*_	714.2**	122.9**	1.614**	0.061**	0.430**	0.670**	0.031**
*V*_*P*_	3015.8+	635.9+	11.567	0.225	0.762	2.223+	0.086**
*V*_*A*_*/V*_*p*_	0.330**	0.334**	0.310**	0.090**	0.270**	0.079**	0.080**
*V*_*D*_*/V*_*p*_	0.000	0.000	0.000	0.639**	0.000	0.620**	0.558**
*V*_*M*_*/V*_*p*_	0.433**	0.473**	0.551**	0.000	0.166**	0.000	0.000
*V*_*e*_*/V*_*p*_	0.237**	0.193**	0.140**	0.271**	0.564**	0.301**	0.362**
*H*_*N*_^*2*^	0.330**	0.334**	0.310**	0.090**	0.270**	0.079**	0.080**
*H*_*B*_^*2*^	0.330**	0.334**	0.310**	0.729**	0.270**	0.699**	0.638**

*V*_*A*_ additive variance, *V*_*D*_ dominance variance, *V*_*M*_, maternal variance, *V*_*E*_ environmental variance, *V*_*P*_ phenotypic variance. *SCY* seedcotton yield, *LY* lint yield, *LP* lint percent, *BW* boll weight, *FL* fiber length, *FS* fiber strength, *MIC* micronaire, *GOS* seed gossypol content, *OIL* seed oil content, *PRO* seed protein content

^+^*P<0*.*05*,

**P<0.*001*.

In both F_1_ and F_2_, CRI 44 and CRI 45 had significant and highest positive additive effects for SCY and LY; and NMGA-96 also had positive additive effects for SCY and LY, and effects were significant in F_2_ (Table 9). NMGA-017 had negative additive effect for SCY and LY in F_1_ and F_2_, but only its F_2_ had significantly negative additive effect. NMGA-085 had significantly negative additive effects in SCY and LY in F_1_, while its negative additive effect was not significant in F_2_. NMGA-098 had negative additive effects in both F_1_ and F_2_, and its negative effects in F_2_ were significant. Both NMGA-100 and NMGA-145 had additive effects but insignificant (Table 9).

**Table 9:** Predicted additive effects for five parents for lint yield and yield component traits based on tests on eight parents (six introgression lines and two commercial cultivars) and their 56 F_1_ and F_2_ hybrids.

Parent	SCY	LY	LP	BW	FL	FS	MIC
**F**_**1**_							
NMGA-017	-8.974	-6.249	-1.351**	0.153	0.856**	0.385**	-0.067
NMGA-085	-16.492*	-8.730*	-0.869**	-0.100	-0.176	0.078	0.000
NMGA-096	5.871	4.149	0.901**	0.171+	0.209	0.191	-0.150**
NMGA-098	-4.682	-2.994	-0.541	-0.113	-0.727**	-0.472**	0.121**
NMGA-100	4.338	0.593	-0.722	-0.041	-0.122	-0.255	0.137**
NMGA-145	-8.261	-3.890	-0.145	-0.184	-0.303	0.174	-0.194**
CRI 44	17.475*	10.290+	1.496*	0.180*	0.171	-0.158	-0.012
CRI 45	10.725	6.831	1.230**	-0.067	0.092	0.056	0.165**
**F**_**2**_							
NMGA-017	-12.551	-6.047+	-0.681+	0.122	0.588*	0.538	-0.034
NMGA-085	-1.657	-2.082	-0.976*	-0.049	-0.137	-0.059	0.031+
NMGA-096	-28.313**	-10.859**	0.668*	0.121*	0.317*	0.169	-0.102
NMGA-098	-16.349*	-7.390*	-0.702+	-0.104+	-0.381**	-0.369+	0.086
NMGA-100	-2.644	-2.880	-1.347**	-0.059	-0.017	-0.186	0.044
NMGA-145	-5.093	-2.780	-0.612+	-0.094	-0.327+	-0.076	-0.037+
CRI 44	31.467**	16.541**	2.643**	0.114	0.000	-0.249	-0.016
CRI 45	35.142**	15.496**	1.007**	-0.050	-0.044	0.231	0.026*
Correl (F1/P)	0.661	0.534	0.928**	0.871**	0.874**	0.868**	0.930**
Correl (F2/P)	0.904**	0.903**	0.944**	0.903**	0.900**	0.889**	0.905**
Correl (F_1_/F_2_)	0.564	0.434	0.900**	0.975**	0.938**	0.863**	0.830*

*SCY* seedcotton yield, *LY* lint yield, *LP* lint percent, *BW* boll weight, *FL* fiber length, *FS* fiber strength, *MIC* micronaire, *GOS* seed gossypol content, *OIL* seed oil content, *PRO* seed protein content

^+^*P<0*.*05*,

*P<0.*01*,

***P<0.*01*.

Both CRI 44 and CRI 45 had significant and positive additive effects for LP in both generations, while CRI 44 also had positive additive effect for boll weight (BW), and CRI had significant and positive additive effect for micronaire (MIC) in both generations (Table 9). Among the six ILs, NMGA-017 and NMGA-085 had significant negative additive effects for LP in both generations; NMGA-098, NMGA-100, and NMGA-145 had significant negative additive effects in F_2_; and only NMGA-096 had significant positive additive effect for LP and BW in both generations.

For fiber quality traits, NMGA-017 had significant positive additive effects for fiber length (FL) and fiber strength (FS) in both generations, while NMGA-098 had significant negative additive effects for FL in F_2_ and for FS in F_1_ and F_2_ (Table 9). Both NMGA-096 and NMGA-145 had significant negative additive effects for MIC (i.e., in reducing MIC), while NMGA-098 and NMGA-100 had significant positive additive effects for MIC in F_1_ (Table 9).

Correlations between F_1_ and F_2_ in additive effects were positive and significant for all the traits except for SCY and LY (Table 9), indicating an overall congruence of additive effects between the two generations. The additive effects in both generations were positively and significantly correlated with the parent performance for all the traits except for SCY and LY in F_1_ (Table 9). The results indicate that additive effects were predominantly determined by parents’ performance per se. Therefore, parents’ trait performance is very important in selection for high additive effects to enhance trait performance in their progeny including F_1_.

As shown in S3 Table, the predicted additive effects based on the ADM model were highly significantly and positively correlated with GCA for each traits (r = 0.89725 to 0.98757 in F_1_ and r = 0.99946 to 0.99986 in F_2_, *P<0*.*01*) except for FS in F_1_ at 0.74926 (*P*<0.05). The results indicate that the additive effects and GCA effects estimated by the two methods were essentially the same.

Homozygous dominance effects for parents and heterozygous dominance effects for hybrids were further predicted using the MIQNUE-based ADM model (S3 Table). All the parents had significant negative dominant effects for SCY and LY, while NMGA-098 and NMGA-100 had significant positive dominant effects for LP, and the reverse was true for four other ILs. NMGA-085 had consistent negative dominant effect for BW in both generations, while the dominant effects for BW were opposite in the two generations from NMGA-017 and CRI 45. The dominant effects in BW for other parents were detected in only one generation.

NMGA-085 and CRI 44 had significant negative dominant effects for FL and FS in F_1_, while NMGA-017 had consistent positive dominant effects for FS in both generations, and the opposite was true for NMGA-085, NMGA-098, and NMGA-100 which had significant negative dominant effects for FS in F_1_ and F_2_. In F_2_, NMGA-017, NMGA-096, and NMGA-145 had negative dominance effects for MIC, while NMGA-098 and CRI 45 had positive dominant effects for MIC.

Of the 28 cross combinations, 12 hybrids had significant heterozygous dominant effects for SCY and LY, and most of them involved parents NMGA-100, CRI 44 and CRI 45. Of the 12 hybrids, 3 had significant negative effects, while F_1_ from NMGA-096 × CRI 45, NMGA-098 × CRI 45, and NMGA-100 × CRI 45 had the highest positive effects. Consistently, six of these 12 hybrids also had significant heterozygous dominant effects with the same directions of the effects for LP; Of a total of 15 hybrids with significant heterozygous dominant effects for LP, 6 involved parent CRI 44 (in 6 hybrids), followed by parents NMGA-098, NMGA-100, and CRI 45 (each in 4–5 hybrids); three (NMGA-096 × CRI 44, NMGA-096 × CRI 45, and NMGA-100 × CRI 44) had the highest positive heterozygous effects, while another three (NMGA-017 × NMGA-100, NMGA-098 × NMGA-100, and CRI 44 × CRI 45) had the highest negative effects. For BW, 10 and 4 hybrids had significant heterozygous dominant effects in F_1_ (tested in 2007) and F_2_ (tested in 2008), respectively, but the effects were not consistent between the two generations (r = -0.236, *P>0*.*05*).

For FL, only two F_1_ (NMGA-085 × NMGA—098 and CRI 44 × CRI 45) had significant positive heterozygous dominant effects. Of the 15 F_1_ and 11 F_2_ hybrids with significant heterozygous dominant effects for FS, the effects from three hybrids were consistent in both generations, while other four had significant but opposite effects. Therefore, no correlation between F_1_ and F_2_ was detected. For MIC, none of the F_1_ hybrids had significant dominant effects. In F_2_, 9 hybrids had significant heterozygous dominant effects, 3–4 of which involved parents NMGA-085, NMGA-144 and CRI 45. The correlation in the dominant effects between F_2_ and F_3_ was significant (r = 0.392, *P<0*.*01*).

## Discussion and Conclusions

This study involved six introgression lines (ILs) developed from interspcific crossing and backcrossing between Upland cotton (*G*. *hirsutum*) and *G*. *barbadense*. Significant germplasm introgression was introduced to broaden and diversify desirable genes and alleles in Upland cotton, as evident in marker analysis and quantitative trait mapping [13]. This report represents one of the first studies to investigate breeding potentials of a set of ILs using a full diallel analysis. The results showed that an increased mid-parent heterosis was manifested in several hybrids between ILs and a commercial cultivar, which also out-yielded the high-yielding cultivar in F_1_, F_2_ and F_3_ generations. A further analysis indicated that general combining ability (GCA) variance is predominant for all the traits, while specific combining ability (SCA) variance was either non-existent or much lower than GCA. Since GCA is controlled by additive effects, an ADM model was employed to predict the effects. Not surprisingly, the estimated GCA effects and predicted additive effects were highly significantly and positively correlated. Furthermore, GCA and additive effects for each trait were also significantly and positively correlated among generations, suggesting that F_2_ and F_3_ generations can be used to analyze combining abilities and estimate genetic parameters as a proxy to F_1_.

The SCA effects were often detected predominant in many previous studies [28–42]. However, in this study, variances for GCA were either the only significant effects or higher than SCA when SCA effects existed; and GCA × E were very lower or nonexistent, while SCA × E were not detected. The results indicated that hybrid performance in this set of 8 × 8 diallel crosses including 6 ILs and 2 commercial cultivars was predominantly determined by GCA effects or additive effects. Therefore, the use of interspecific ILs may have rendered different results in combining ability and genetic analysis from these using intraspecific Upland cotton lines. While it is not unexpected that ILs are inferior to commercial cultivars in yield potentials as demonstrated in this study, some cross combinations with commercial cultivars significantly increased lint yield, indicating that new yield-enhancing gene alleles were introduced to Upland cotton. Further studies of these crosses may uncover these desirable gene alleles for cross breeding in cotton.

This study further showed that mid-parent heterisis (MPH) in lint yield was correlated with MPH for lint percent, and seed oil and protein contents, suggesting positive or negative contributions to yield MPH from MPH of these traits. Many previous studies further showed that the number of bolls plays a major role in yield heterosis [17, 43, 44]. This study also provides another line of evidence that performances among F_1_, F_2_ and F_3_ were highly positively correlated in all the traits evaluated. However, the mid-parent heterosis (MPH) was usually not correlated among the three generations, indicating that non-additive effects may still play an important role leading to variable inbreeding depressions among hybrids. This suggests that a hybrid with the highest heterosis in yield in F_1_ may not retain the yield potential in F_2_ or F_3_.

## Supporting Information

S1 Table Specific combining ability (SCA) for yield, yield component traits, and fiber and seed quality traits in reciprocal crosses of eight Upland cotton lines based on a full diallel of 56 F_1_, F_2_ and F_3_ hybrids (*Gossypium hirsutum*). (XLSX)

S2 Table Useful heterosis of lint yield (LY, kg ha^-1^) In F_1_ (AY, 2007), F_2_ (AY, 2008), and F_3_ (AY, 2009; SD, 2009; and SQ, 2009), as compared with the highest yielding parent (CRI 44), a commercial cultivar. (DOCX)

S3 Table Predicted (homozygous for parents and heterozygous for hybrids) dominance effects for lint yield, yield component and fiber quality traits based on tests on eight parents (six introgression lines and two commercial cultivars) and their 56 F_1_ and F_2_ hybrids. (DOCX)
