# Supplementary material for: Breeding Potential of Introgression Lines Developed from Interspecific Crossing between Upland Cotton (Gossypium hirsutum) and Gossypium barbadense: Heterosis, Combining Ability and Genetic Effects
Source: PLoS One. 2016 Jan 5;11(1):e0143646. doi: 10.1371/journal.pone.0143646 (PMC4701505; doi:10.1371/journal.pone.0143646)
Supplement: S2 Table — (DOCX) [file pone.0143646.s002.docx]

**Supplementary Table 2. Useful heterosis of lint yield (LY, kg ha^-1^) In F_1_ (AY, 2007), F_2_ (AY, 2008), and F_3_ (AY, 2009; SD, 2009; and SQ, 2009), as compared with the highest yielding parent (CRI 44), a commercial cultivar.**

|  |  | **F_1_** |  |  | **F_2_** |  |  |  |  |  | **F_3_** |  |  |  |  |
| --- | --- | --- | --- | --- | --- | --- | --- | --- | --- | --- | --- | --- | --- | --- | --- |
| **Genotype** | **AY 07** | **AY 07** | **AY 07** | **AY 08** | **AY 08** | **AY 08** | **AY 09** | **AY 09** | **AY 09** | **SD 09** | **SD 09** | **SD 09** | **SQ 09** | **SQ 09** | **SQ 09** |
|  | **LY** | **Het** | **R** | **LY** | **Het** | **R** | **LY** | **Het** | **R** | **LY** | **Het** | **R** | **LY** | **Het** | **R** |
| CRI 44 × NMGA-096 | 113.8 | 34.2 | 1 | 53.9 | -26.6 | 31 | 82.4 | 19.3 | 3 | 271.0 | 3.5 | 19 | 98.6 | 2.5 | 11 |
| NMGA-100 × CRI 44 | 113.4 | 33.7 | 2 | 65.3 | -11.1 | 11 | 77.1 | 11.6 | 8 | 311.2 | 18.9 | 2 | 99.7 | 3.6 | 8 |
| CRI 44 × NMGA-100 | 106.2 | 25.3 | 3 | 80.1 | 9.0 | 3 | 73.9 | 6.9 | 13 | 262.7 | 0.3 | 31 | 103.8 | 7.8 | 3 |
| CRI 45 × NMGA-098 | 102.5 | 20.9 | 4 | 59.9 | -18.5 | 20 | 73.8 | 6.8 | 14 | 284.1 | 8.5 | 11 | 101.4 | 5.4 | 4 |
| NMGA-096 × CRI 45 | 102.0 | 20.3 | 5 | 68.8 | -6.4 | 9 | 76.1 | 10.2 | 10 | 294.0 | 12.3 | 8 | 87.3 | -9.3 | 27 |
| CRI 45×NMGA-100 | 101.9 | 20.2 | 6 | 63.5 | -13.6 | 13 | 78.8 | 14.0 | 6 | 260.1 | -0.7 | 32 | 97.2 | 1.0 | 12 |
| CRI 45 × NMGA-096 | 100.0 | 18.0 | 7 | 60.2 | -18.2 | 19 | 76.6 | 10.9 | 9 | 299.9 | 14.5 | 5 | 98.7 | 2.5 | 10 |
| CRI 45 × NMGA-145 | 99.3 | 17.1 | 8 | 59.9 | -18.6 | 21 | 68.5 | -0.9 | 26 | 257.3 | -1.7 | 34 | 100.3 | 4.2 | 7 |
| NMGA-100 × CRI 45 | 98.3 | 15.9 | 9 | 60.2 | -18.1 | 18 | 81.5 | 17.9 | 4 | 322.8 | 23.3 | 1 | 91.9 | -4.5 | 21 |
| NMGA-145 × CRI 44 | 95.9 | 13.1 | 10 | 75.2 | 2.3 | 6 | 72.5 | 5.0 | 17 | 264.7 | 1.1 | 25 | 93.2 | -3.2 | 18 |
| NMGA-017 × CRI 44 | 94.2 | 11.1 | 11 | 55.3 | -24.8 | 26 | 66.1 | -4.3 | 31 | 277.9 | 6.1 | 13 | 117.0 | 21.6 | 1 |
| NMGA-098 × CRI 44 | 94.2 | 11.1 | 12 | 67.4 | -8.3 | 10 | 85.5 | 23.8 | 1 | 304.2 | 16.2 | 4 | 105.9 | 10.1 | 2 |
| NMGA-085 × CRI 44 | 94.0 | 10.8 | 13 | 62.5 | -14.9 | 14 | 70.3 | 1.7 | 23 | 295.3 | 12.8 | 7 | 100.7 | 4.6 | 5 |
| CRI 44 × NMGA-145 | 93.3 | 10.1 | 14 | 78.1 | 6.3 | 4 | 75.3 | 9.0 | 12 | 291.4 | 11.3 | 9 | 95.1 | -1.1 | 15 |
| CRI 44 × NMGA-098 | 91.2 | 7.5 | 15 | 61.5 | -16.4 | 16 | 75.7 | 9.5 | 11 | 269.7 | 3.0 | 20 | 98.8 | 2.7 | 9 |
| NMGA-098 × CRI 45 | 89.6 | 5.7 | 16 | 61.9 | -15.8 | 15 | 69.2 | 0.2 | 25 | 273.0 | 4.3 | 16 | 79.4 | -17.5 | 40 |
| CRI 44 × CRI 45 | 89.5 | 5.6 | 17 | 91.9 | 25.0 | 1 | 71.7 | 3.8 | 19 | 264.6 | 1.1 | 27 | 92.7 | -3.6 | 20 |
| NMGA-145 × CRI 45 | 89.3 | 5.3 | 18 | 69.7 | -5.2 | 8 | 71.5 | 3.5 | 20 | 277.5 | 6.0 | 14 | 92.9 | -3.5 | 19 |
| NMGA-017 × CRI 45 | 88.5 | 4.4 | 19 | 56.2 | -23.5 | 24 | 58.8 | -14.9 | 44 | 265.6 | 1.4 | 24 | 93.7 | -2.7 | 17 |
| CRI 44 × NMGA-017 | 88.5 | 4.3 | 20 | 60.2 | -18.1 | 17 | 77.7 | 12.4 | 7 | 263.7 | 0.7 | 29 | 83.9 | -12.8 | 34 |
| NMGA-085 × NMGA-096 | 88.2 | 4.0 | 21 | 39.8 | -45.8 | 46 | 59.2 | -14.2 | 42 | 237.0 | -9.5 | 43 | 86.5 | -10.1 | 30 |
| NMGA-145 × NMGA-096 | 88.0 | 3.7 | 22 | 51.9 | -29.4 | 34 | 64.5 | -6.6 | 32 | 224.0 | -14.5 | 48 | 79.3 | -17.6 | 42 |
| NMGA-100 × NMGA-096 | 87.4 | 3.1 | 23 | 36.6 | -50.2 | 48 | 59.4 | -14.0 | 41 | 263.5 | 0.6 | 30 | 80.6 | -16.2 | 38 |
| NMGA-085 × NMGA-098 | 84.3 | -0.6 | 24 | 41.0 | -44.2 | 43 | 56.6 | -18.1 | 46 | 207.2 | -20.8 | 53 | 76.5 | -20.5 | 45 |
| CRI 45 × CRI 44 | 84.2 | -0.7 | 25 | 83.5 | 13.6 | 2 | 71.3 | 3.2 | 21 | 307.3 | 17.4 | 3 | 91.1 | -5.3 | 22 |
| CRI 45 × NMGA-085 | 82.5 | -2.8 | 26 | 75.6 | 2.9 | 5 | 69.7 | 0.9 | 24 | 285.1 | 8.9 | 10 | 87.9 | -8.7 | 24 |
| NMGA-145 × NMGA-017 | 82.3 | -2.9 | 27 | 54.1 | -26.4 | 30 | 67.2 | -2.7 | 29 | 247.9 | -5.3 | 37 | 74.5 | -22.5 | 49 |
| NMGA-085 × NMGA-017 | 81.7 | -3.7 | 28 | 54.9 | -25.4 | 27 | 67.7 | -2.0 | 28 | 232.3 | -11.3 | 44 | 87.8 | -8.7 | 25 |
| CRI 44 × NMGA-085 | 81.3 | -4.1 | 29 | 54.7 | -25.6 | 28 | 83.4 | 20.7 | 2 | 268.0 | 2.3 | 22 | 94.0 | -2.3 | 16 |
| CRI 45 × NMGA-017 | 79.9 | -5.7 | 30 | 70.2 | -4.5 | 7 | 73.4 | 6.3 | 15 | 248.4 | -5.1 | 36 | 97.2 | 1.0 | 13 |
| NMGA-017 × NMGA-096 | 79.2 | -6.6 | 31 | 40.9 | -44.4 | 44 | 55.3 | -20.0 | 51 | 196.1 | -25.1 | 56 | 82.7 | -14.1 | 37 |
| NMGA-096 × NMGA-100 | 77.2 | -9.0 | 32 | 34.0 | -53.8 | 52 | 64.0 | -7.4 | 33 | 264.5 | 1.0 | 28 | 68.0 | -29.3 | 56 |
| NMGA-085 × CRI 45 | 76.3 | -10.1 | 33 | 64.4 | -12.4 | 12 | 71.9 | 4.1 | 18 | 277.3 | 5.9 | 15 | 85.2 | -11.4 | 33 |
| NMGA-098 × NMGA-096 | 76.2 | -10.2 | 34 | 35.2 | -52.1 | 50 | 60.1 | -13.0 | 38 | 266.0 | 1.6 | 23 | 97.1 | 0.9 | 14 |
| NMGA-100 × NMGA-017 | 75.7 | -10.8 | 35 | 51.3 | -30.2 | 36 | 66.9 | -3.2 | 30 | 282.3 | 7.8 | 12 | 83.6 | -13.1 | 35 |
| NMGA-096 × NMGA-098 | 73.7 | -13.1 | 36 | 33.0 | -55.1 | 55 | 61.5 | -10.9 | 36 | 231.8 | -11.5 | 46 | 74.3 | -22.8 | 51 |
| NMGA-085 × NMGA-145 | 73.6 | -13.2 | 37 | 33.4 | -54.6 | 54 | 55.4 | -19.8 | 50 | 202.2 | -22.8 | 54 | 70.9 | -26.3 | 54 |
| NMGA-145 × NMGA-100 | 73.5 | -13.4 | 38 | 54.4 | -26.0 | 29 | 55.9 | -19.0 | 48 | 198.6 | -24.2 | 55 | 74.9 | -22.2 | 48 |
| NMGA-096 × CRI 44 | 73.4 | -13.5 | 39 | 53.2 | -27.7 | 32 | 80.6 | 16.6 | 5 | 298.4 | 14.0 | 6 | 100.5 | 4.4 | 6 |
| NMGA-145 × NMGA-098 | 72.6 | -14.4 | 40 | 50.1 | -31.8 | 39 | 53.2 | -23.0 | 52 | 241.5 | -7.8 | 39 | 87.6 | -9.0 | 26 |
| NMGA-096 × NMGA-085 | 71.8 | -15.3 | 41 | 52.5 | -28.6 | 33 | 60.0 | -13.1 | 39 | 221.3 | -15.5 | 49 | 76.4 | -20.6 | 46 |
| NMGA-098 × NMGA-100 | 71.2 | -16.1 | 42 | 51.3 | -30.2 | 35 | 48.6 | -29.7 | 55 | 239.5 | -8.5 | 41 | 90.9 | -5.5 | 23 |
| NMGA-145 × NMGA-085 | 70.5 | -16.9 | 43 | 58.8 | -20.1 | 23 | 56.9 | -17.6 | 45 | 209.1 | -20.1 | 52 | 79.6 | -17.3 | 39 |
| NMGA-096 × NMGA-145 | 70.4 | -17.0 | 44 | 36.2 | -50.7 | 49 | 61.9 | -10.4 | 35 | 239.1 | -8.7 | 42 | 77.8 | -19.2 | 44 |
| NMGA-100 × NMGA-098 | 69.9 | -17.5 | 45 | 45.2 | -38.5 | 41 | 56.3 | -18.6 | 47 | 217.7 | -16.9 | 51 | 78.5 | -18.4 | 43 |
| NMGA-017 × NMGA-145 | 69.9 | -17.6 | 46 | 26.0 | -64.6 | 56 | 70.4 | 1.9 | 22 | 271.7 | 3.8 | 17 | 79.3 | -17.6 | 41 |
| NMGA-017 × NMGA-100 | 69.2 | -18.4 | 47 | 37.0 | -49.7 | 47 | 73.4 | 6.2 | 16 | 271.4 | 3.7 | 18 | 85.8 | -10.8 | 32 |
| NMGA-085 × NMGA-100 | 68.7 | -19.0 | 48 | 43.8 | -40.4 | 42 | 59.2 | -14.3 | 43 | 229.8 | -12.2 | 47 | 71.8 | -25.4 | 53 |
| NMGA-098 × NMGA-145 | 66.4 | -21.7 | 49 | 40.3 | -45.2 | 45 | 59.7 | -13.6 | 40 | 247.2 | -5.6 | 38 | 74.1 | -23.0 | 52 |
| NMGA-098 × NMGA-085 | 63.3 | -25.3 | 50 | 50.1 | -31.8 | 38 | 67.9 | -1.8 | 27 | 239.9 | -8.4 | 40 | 86.7 | -9.9 | 29 |
| NMGA-100 × NMGA-145 | 62.8 | -26.0 | 51 | 56.2 | -23.5 | 25 | 45.5 | -34.2 | 56 | 218.1 | -16.7 | 50 | 75.1 | -22.0 | 47 |
| NMGA-096 × NMGA-017 | 61.4 | -27.6 | 52 | 33.4 | -54.6 | 53 | 63.4 | -8.2 | 34 | 264.6 | 1.1 | 26 | 83.3 | -13.5 | 36 |
| NMGA-100 × NMGA-085 | 61.1 | -28.0 | 53 | 49.2 | -33.1 | 40 | 49.4 | -28.4 | 54 | 249.2 | -4.8 | 35 | 74.4 | -22.7 | 50 |
| NMGA-017 × NMGA-085 | 59.9 | -29.3 | 54 | 50.2 | -31.7 | 37 | 55.7 | -19.3 | 49 | 232.1 | -11.4 | 45 | 86.8 | -9.8 | 28 |
| NMGA-098 × NMGA-017 | 57.4 | -32.3 | 55 | 58.8 | -20.1 | 22 | 61.4 | -11.1 | 37 | 269.3 | 2.8 | 21 | 85.9 | -10.7 | 31 |
| NMGA-017 × NMGA-098 | 53.6 | -36.8 | 56 | 34.2 | -53.4 | 51 | 50.8 | -26.5 | 53 | 259.5 | -0.9 | 33 | 70.2 | -27.0 | 55 |
| NMGA-017 | 48.0 | -43.4 |  | 38.5 | -47.6 |  | 49.7 | -28.1 |  | 239.6 | -8.5 |  | 76.4 | -20.6 |  |
| NMGA-085 | 54.4 | -35.9 |  | 43.5 | -40.8 |  | 44.5 | -35.6 |  | 226.4 | -13.5 |  | 71.0 | -26.2 |  |
| NMGA-096 | 52.6 | -38.0 |  | 30.1 | -59.1 |  | 51.5 | -25.4 |  | 233.9 | -10.7 |  | 78.9 | -18.0 |  |
| NMGA-098 | 56.9 | -32.9 |  | 26.3 | -64.2 |  | 44.9 | -35.1 |  | 217.8 | -16.8 |  | 81.2 | -15.6 |  |
| NMGA-100 | 69.0 | -18.6 |  | 39.5 | -46.3 |  | 48.3 | -30.1 |  | 182.5 | -30.3 |  | 64.7 | -32.7 |  |
| NMGA-145 | 59.0 | -30.4 |  | 32.9 | -55.2 |  | 61.5 | -11.0 |  | 214.1 | -18.2 |  | 77.6 | -19.4 |  |
| CRI 44 | 84.8 | 0.0 |  | 73.5 | 0.0 |  | 69.1 | 0.0 |  | 261.8 | 0.0 |  | 96.2 | 0.0 |  |
| CRI 45 | 71.3 | -15.9 |  | 65.2 | -11.3 |  | 64.2 | -7.0 |  | 262.5 | 0.3 |  | 85.8 | -10.9 |  |

*Het* useful heterosis, *R* rank, *AY* Anyang, *SD* Shandong, *SQ* Shangqiu
