# Supplementary material for: Breeding Potential of Introgression Lines Developed from Interspecific Crossing between Upland Cotton (Gossypium hirsutum) and Gossypium barbadense: Heterosis, Combining Ability and Genetic Effects
Source: PLoS One. 2016 Jan 5;11(1):e0143646. doi: 10.1371/journal.pone.0143646 (PMC4701505; doi:10.1371/journal.pone.0143646)
Supplement: S3 Table — (DOCX) [file pone.0143646.s003.docx]

S**upplementary Table 3. Predicted (homozygous for parents and heterozygous for hybrids) dominance effects for lint yield, yield component and fiber quality traits based on tests on eight parents (six introgression lines and two commercial cultivars) and their 56 F_1_ and F_2_ hybrids.**

| **Genotype** | **SCY** | **LY** | **LP** | **BW** |  | **FL** | **FS** |  | **MIC** |  |
| --- | --- | --- | --- | --- | --- | --- | --- | --- | --- | --- |
|  | **F_1_** | **F_1_** | **F_1_** | **F_1_** | **F_2_** | **F_1_** | **F_1_** | **F_2_** | **F_1_** | **F_2_** |
| NMGA-017 (P1) | -29.871* | -12.045* | -0.294+ | -0.264* | 0.545+ | -0.154 | 0.221* | 2.493+ | -0.023 | -0.457* |
| NMGA-085 (P2) | -23.232* | -10.694* | -0.691* | -0.225* | -0.377+ | -0.239+ | -0.038** | -0.941+ | 0.008 | 0.139* |
| NMGA-096 (P3) | -40.667* | -17.628* | -0.143 | -0.261* | 1.264 | -0.337 | -0.055* | 0.285 | -0.005 | -0.285** |
| NMGA-098(P4) | -24.740* | -8.991* | 0.460* | 0.016 | -0.737+ | -0.610 | -0.218* | -2.266+ | -0.003 | 0.246+ |
| NMGA-100 (P5) | -24.062* | -8.009* | 0.719* | -0.594* | -0.132 | -0.322 | -0.220* | -0.835* | 0.019 | 0.088 |
| NMGA-145 (P6) | -29.552* | -11.582* | 0.185 | -0.321* | 0.158 | -0.232 | -0.017* | 1.193+ | -0.028 | -0.302* |
| CRI 44 (P7) | -24.240* | -12.775* | -0.929* | -0.236* | 0.067 | -0.374+ | -0.037* | -0.425 | -0.022 | -0.060 |
| CRI 45 (P8) | -41.016* | -17.797* | -0.172* | -0.253* | 0.173+ | -0.583 | -0.044* | 0.116 | 0.012 | 0.329* |
| P1 × P2 | 8.646 | 4.601 | 0.461** | 0.189 | 0.485 | -0.008 | -0.030+ | -2.184 | 0.000 | 0.204 |
| P1 × P3 | 2.205 | 0.775 | 0.031 | -0.063 | -0.434+ | 0.373 | 0.041 | 0.209 | -0.001 | 0.215 |
| P1 × P4 | -21.448+ | -9.758+ | -0.628 | -0.001 | -0.049 | 0.275 | 0.046* | -0.426 | -0.002 | 0.274 |
| P1 × P5 | 9.633+ | 0.916 | -0.893** | 0.238* | -0.148 | 0.232 | 0.008 | 2.190+ | 0.002 | -0.409+ |
| P1 × P6 | 19.588* | 6.837+ | -0.225 | 0.096+ | 0.175 | -0.273 | -0.127* | -1.510* | 0.021 | 0.363+ |
| P1 × P7 | 16.820* | 8.328+ | 0.677 | 0.026 | 0.478 | -0.280 | -0.191* | 0.999+ | 0.014 | 0.078 |
| P1 × P8 | 12.777 | 4.964 | 0.100 | 0.214** | -0.466 | 0.683 | -0.033* | 0.418 | 0.002 | -0.094 |
| P2 × P3 | 19.950** | 7.677* | -0.096 | 0.204+ | 0.063 | 0.062 | 0.127* | 1.210* | -0.007 | 0.182** |
| P2 × P4 | 14.885 | 5.086 | -0.292+ | -0.115+ | 0.006 | 0.266+ | -0.014 | 0.575 | 0.014 | -0.114** |
| [2 × P5 | -20.182+ | -8.274+ | -0.044 | -0.013 | -0.040 | -0.017 | 0.021+ | -0.341 | -0.016 | 0.053 |
| P2 × P6 | 1.118 | 0.924 | 0.263 | -0.063 | -0.267 | -0.069 | -0.063* | 1.525 | -0.014 | -0.105 |
| P2 × P7 | 3.891 | 2.727 | 0.478* | 0.064 | 0.079 | 0.244 | 0.087* | -0.890 | 0.013 | 0.158+ |
| P2 × P8 | -3.017 | -1.728 | -0.073 | 0.073 | -0.023 | -0.142 | -0.020+ | 1.473* | -0.007 | -0.390** |
| P3 × P4 | 8.645 | 2.828 | -0.281* | 0.046 | -0.083 | -0.121 | -0.097* | -1.152+ | 0.003 | -0.129 |
| P3 × P5 | 13.356* | 4.627+ | -0.279 | 0.431** | 0.123 | 0.208 | 0.099 | -0.838 | -0.012 | -0.162 |
| P3 × P6 | 11.805+ | 4.238 | -0.317 | 0.142+ | -0.624+ | 0.431 | 0.140* | 0.386 | -0.010 | -0.116 |
| P3 × P7 | 4.280 | 4.932 | 1.096** | 0.040 | -0.183 | -0.117 | 0.025 | -0.263 | 0.002 | -0.175+ |
| P3 × P8 | 28.631* | 15.110* | 0.843* | -0.086 | -0.267 | 0.007 | -0.148 | 1.350+ | 0.013 | -0.099 |
| P4 × P5 | -1.357 | -3.130 | -0.784** | 0.006 | 0.353 | -0.058 | 0.097* | 0.454 | -0.003 | 0.053 |
| P4 ×P6 | -3.952 | -1.554 | 0.065 | -0.168 | 0.120 | -0.079 | 0.307* | 0.018 | -0.008 | 0.094 |
| P4 × P7 | 14.609 | 7.271+ | 0.449* | 0.041 | 0.244+ | 0.337 | -0.087* | 1.136+ | 0.015 | -0.132 |
| P4 × P8 | 32.087** | 13.680** | 0.123 | 0.033 | -0.085 | 0.009 | -0.010 | 0.716 | 0.005 | 0.181 |
| P5 × P6 | -10.479 | -7.925 | -1.402** | 0.149+ | -0.351+ | -0.131 | 0.012 | -1.540* | 0.017 | 0.346+ |
| P5 × P7 | 37.086* | 18.138** | 0.819+ | 0.359** | -0.316 | 0.117 | -0.125+ | 0.541 | 0.001 | 0.115 |
| P5 × P8 | 25.635** | 12.371** | 0.576** | -0.026 | 0.091 | 0.193 | 0.224* | -0.415 | -0.007 | 0.202 |
| P6 × P7 | 12.479* | 6.545* | 0.453+ | 0.099 | 0.216 | 0.217 | 0.019 | -1.500+ | 0.013 | -0.054 |
| P6 × P8 | 17.938 | 9.476+ | 0.680* | 0.180** | -0.459 | 0.121 | -0.184* | -0.423 | 0.009 | -0.236+ |
| P7 × P8 | -18.248 | -10.161 | -0.934* | 0.045 | 0.401 | 0.369+ | 0.282* | -1.338* | -0.016 | 0.000 |

*SCY* seedcotton yield, *LY* lint yield, *LP* lint percent, *BW* boll weight, *FL* fiber length, *FS* fiber strength, *MIC* micronaire

^+^, * and ** Significant at P=0.05, 0.01, and 0.001 levels, respectively.
